# Robotic right colectomy in a patient with ventriculoperitoneal shunt. Report of a case

**DOI:** 10.1016/j.ijscr.2019.05.018

**Published:** 2019-05-11

**Authors:** Igor Monsellato, Marco Lodin, Fabio Priora

**Affiliations:** SS. Antonio e Biagio e cesare Arrigo Hospital, Alessandria, Italy

**Keywords:** Robotic surgery, Ventriculoperitoneal shunt, Robotic right colectomy, Colorectal cancer, Minimally-invasive surgery, Hydrocephalus

## Abstract

Introduction: Ventriculoperitoneal shunt procedure has become the most common neurosurgical method for hydrocephalus because it considerably improves patients prognosis.

Pneumoperitoneum has been considered a contraindication to laparoscopic surgery because of risk for increased intracranial pressure during pneumoperitoneum and/or malfunction/infection of the VP shunt itself. Laparoscopic resection of the cecum and of the rectum for cancer has been reported.

Presentation of case: A 74-year old man with ventriculoperitoneal shunt for normal pressure hydrocephalus referred to the emergency medicine ward for COPD, lower limb oedema and severe anemia. CT Scan showed a substenotic tumor of the right colon and non-specific enlarged regional lymph nodes, with no distant metastases. Colonoscopy confirmed the presence of an ulcerated tumor of the right colon involving half of the colic lumen. A right colectomy with CME was carried out, with a stapled intracorporeal ileocolic side-to-side isoperistaltic anastomosis and without manipulating the VPS catheter.

Discussion: Laparoscopic surgery in patients with VPS tubes was previously contraindicated because of the possibility of shunt-associated complications, that may include shunt malfunction due to increased intra-abdominal pressure, damage or infection of the catheter. Some authors reported that intracranial pressure increased up to 25 mmHg at a pneumoperitoneum pressure of 12 mmHgHerein we report, to our knowledge, the first case report of robotic assisted right colectomy for cancer in a patient with a VP shunt.

Conclusion: Robotic assistance may allow to perform colorectal resection safely and with low risk also in patients with ventriculoperitoneal shunt.

## Highlights

• Laparoscopic surgery in patients with VPS tubes was previously contraindicated.
• Robotic right colectomy is associated with improved recovery.
• No need of VPS catheter manipulation is needed in robotic surgery.
• The first reported case of a robotic right colectomy with an intracorporeal anastomosis in a patients with a VPS.
• Robotic right colectomy is safe also in patients with VPS, with short hospital stay and fast postoperative recovery.

## 1 Introduction

Ventroculoperitoneal shunt (VPS) insertion is the standard treatment for hydrocephalus that can results from infection, meningitis, brain tumors, head trauma and intracranial haemorrage [1]. Improvement in shunt technology and catheter insertion had led to a growing number of patients with VPS needing abdominal surgery. On the other side, the presence of a VP shunt has been considered in the past a contraindication for minimally-invasive surgery and open laparotomy with externalization of the distal part of the catheter was usually performed to avoid pneumoperitoneum-related complications, such as shunt malfunction and/or infection.

Moreover, subcutaneous emphysema along the catheter tract and shunt failure were also reported [2,3].

Laparoscopic right colectomy with clamping of the VPS and laparoscopic rectal resection without manipulating the catheter have been recently reported [4,5]. Robotic assistance has been considered safe and with peculiar characteristics such as 3D magnified vision, endowrist technology and better ergonomics that overcome pitfalls of standard laparoscopy. Herein, we report the first case, to the best of our knowledge, of a robotic right colectomy for right colon cancer with intracorporeal anastomosis in a patient with a VP shunt. The work has been reported in line with the SCARE criteria [6].

## 2 Presentation of case

**Fig. 1:**
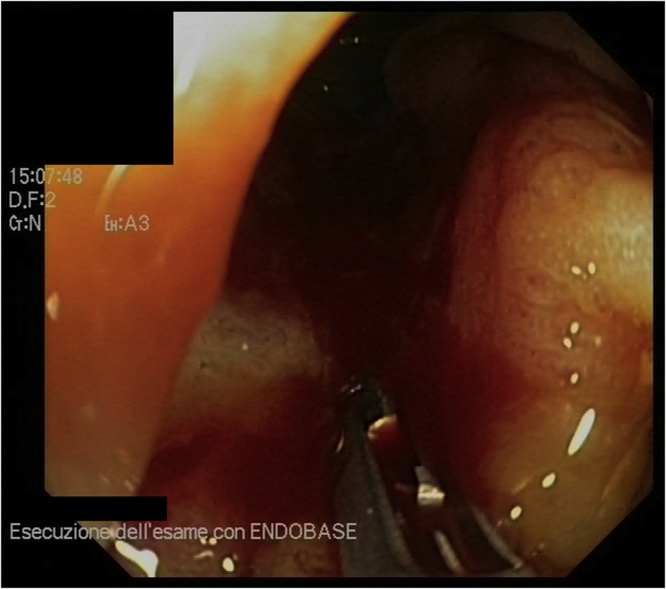
Endoscopical view of the tumor (after biopsy).

**Fig. 2:**
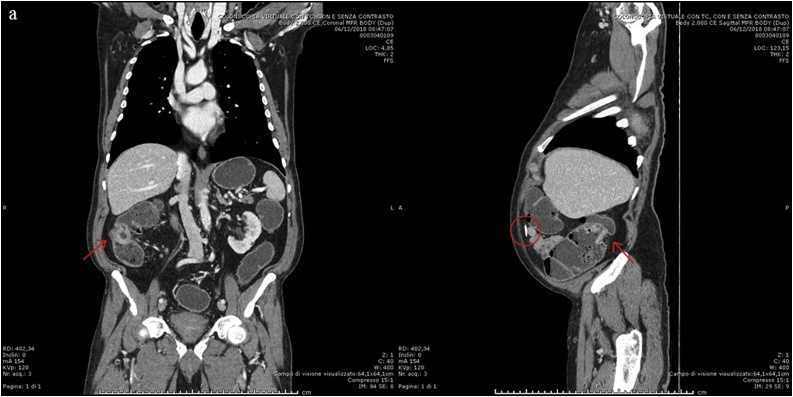
CT Scan showing the tumor of the right colon (arrows). a) Coronal MPR image; b) Sagittal MPR image, in which It is noticeable the VPS Catheter (circle).

**Fig. 3:**
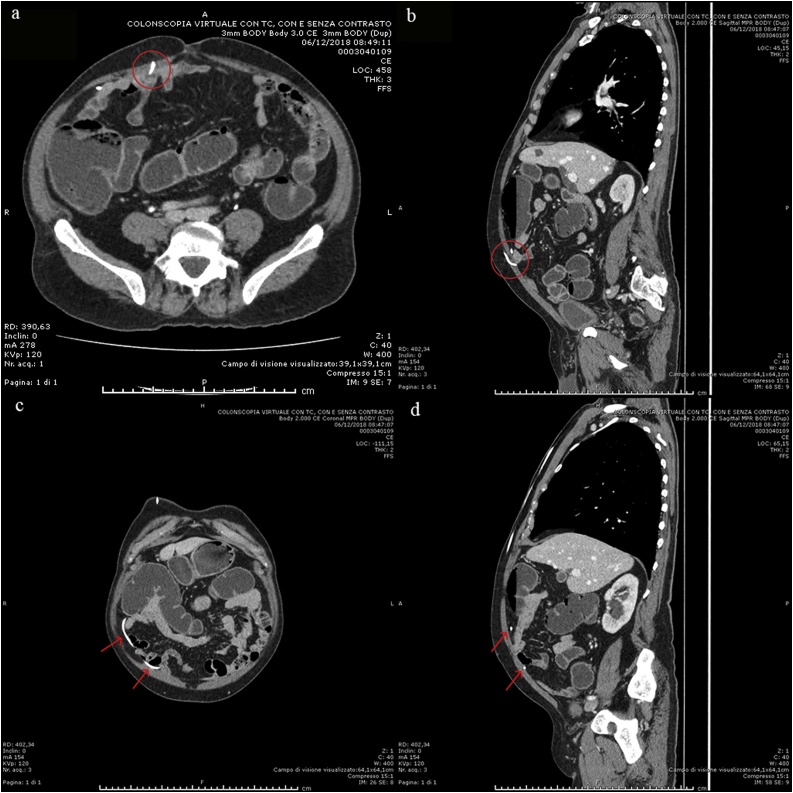
CT Scan showing the site of entrance into the abdomen (circle; a. Coronal view; b. Sagittal MPR image) and its pathway in the abdominal cavity (arrows; c. Coronal MPR image; d. Sagittal MPR image).

A 74-year old man with ventriculoperitoneal shunt for normal pressure hydrocephalus referred to the emergency medicine ward for COPD, lower limb oedema and severe anemia (Hb 5,2 g/dL). A right colon cancer was diagnosed by a colonscopy. He also had diabetes, hypertension, chronic heart failure, and a VPS inserted in 2016 for normal pressure hydrocephalus. Colonoscopy showed an ulcerated tumor of the right colon involving half of the colic lumen (Fig. 1). Histological examination of the biopsy specimen revealed a moderately differentiated adenocarcinoma. After discharge he was referred to the surgical outpatient clinic and underwent a CT Scan that showed a substenotic tumor of the right colon and non-specific enlarged regional lymph nodes, with no distant metastases (Fig. 2a–b). A non-functioning adenoma of the left adrenal gland was also reported. CT scan also showed the VPS catheter routed subcutaneously into the right middle-lower part of the abdomen (Fig. 3a–d). After consultation with a neurosurgeon, no indication to shunt deviation or additional valve insertion was needed. A standard antibiotic preoperative prophylaxis by cephalosporin 2gr and metronidazole 500 mg was administered to the patient 30 min before the procedure started, as usual. Patient was placed in supine position with a slight Trendelemburg (5 °) and a 5 ° left tilt. Penumoperitoneum was established by Veress needle in left hypochondrium and it was maintained at a pressure of 8–10 mmHg. Surgery was performed (I.M.) using a conventional five-trocart robotic technique: a 12 mm laparoscopic trocart was placed in left iliac fossa for robotic endoscope, then three 8 mm robotic trocarts were placed, at first, in the left hypogastrium and below the left rib margin and then in sovrapubic area, under view control after locating the VPS catheter; a 12 mm laparoscopic trocart was placed in the left flank for the assistance, as usual. A laparoscopic exploration of the abdominal cavity was first performed, that showed several visceral adherences and the tumor of the right colon, retracting the serosa. The endoscopic tattoo was also visible. A small umbilical hernia (5 mm) was present and just repaired, laparoscopically. The VPS catheter entered abdominal cavity 3 cm right the umbilicus region with the tip laying down to the pelvis. A sponge was placed in the pelvis to protect VPS catheter. No catheter manipulation was necessary to place the suprapubic robotic trocart. Robot approached surgical table from above, with an angle of 60 °, from patient’s right side. A right colectomy with CME was carried out as usual, with a stapled intracorporeal ileocolic side-to-side isoperistaltic anastomosis. The specimen was inserted into a bag and a Pfannenstiel incision was performed for specimen extraction. Operative time was 280 min with minimal blood loss. Overall pneumoperitoneum time was 190 min. Pathology report showed an ulcerated moderately differentiated adenocarcinoma, with low grade tumor budding, ENVI -, PI -; pT3 pN0, pStage IIA. Antibiotics (cephalosporin 2gr/day) were administered until POD 3. No signs of neurological alterations or systemic and locoregional infection were experienced in the postoperative stay and patient was discharged on POD 5. No need of follow up brain CT Scan was requested by the neurosurgeon in absence of neurological altered signs. Patient was followed up in outpatient clinic on POD 9 and no signs of neurological alterations or infection were observed.

## 3 Discussion

Laparoscopic surgery in patients with VPS tubes was previously contraindicated because of the possibility of shunt-associated complications, that may include shunt malfunction due to increased intra-abdominal pressure, damage or infection of the catheter. Some authors reported that intracranial pressure increased up to 25 mmHg at a pneumoperitoneum pressure of 12 mmHg [7]. Other authors described a subcutaneous emphysema along the catheter tract and a VPS failure [8,9].

Yoshihara et al. reported the feasibility and safety of performing laparoscopic cholecystectomy in patient with VPS, showing also results from seven studies including 14 patients. In two of these reported cases, the shunt catheter was clamped before surgery to prevent shunt malfunction, in one case, the intraperitoneal part of the catheter was moved away from the operating field, while in the remaining cases no specific measures were undertaken to prevent complications [10]. Recently, Matsumoto et al. conducted an experiment on five different programmable valve used at maximum setting and showed that laparoscopic surgery can be performed safely, if detailed information about VPS systems are available and characteristics of VPS valve are taken of [11].

In 2012, Wadhwa et al. stated that the presence of a VP shunt does not pose an increased risk of postoperative complications in patients undergoing gastrectomy or colectomy. The presence of a shunt was associated, indeed, with dense adhesions in 1 (14%) of the 7 patients in their series, but not with an increased risk of postoperative complications [12].

Neal et al. stated that a disruption of the catheter or a reflux of cerebrospinal fluid did not occur until a pressure of 80 mmHg is not reached, therefore a pneumoperitoneum pressure of 8–10 mmHg has been considered safe and with few adverse effects. We maintained a pressure between 8 and 10 mmHg, preserving an adequate surgical field, thanks to the “hang up” effect of the robotic arms, that allows a wider operative field also with low pneumoperitoneum pressure. Two laparoscopic right colectomies in patients with VPS have been recently reported. In both the two cases an extracorporeal anastomosis was fashioned after a laparoscopic dissection. Torigoe et al. performed an enlargement of the umbilical port to 3 cm, while Imagami et al. performed a minilaparotomy to connect the incision of the upper abdomen to the umbilicus and a washing of the abdominal cavity was carried out afterwards [4,13]. Compared to laparoscopic right colectomy, robotic right colectomy was associated with improved recovery bowel function, significantly lowered oral morphine equivalent usage, decreased short-term complications, reduced estimated blood loss [14,15]. In a multicenter propensity score-matched comparison study between robotic and laparoscopic right colectomy, the intracorporeal anastomosis group had significantly longer operative times (p < 0.0001), but lower conversion to open rate (p = 0.01), shorter hospital length of stay (p = 0.02) and lower complication rate from after discharge to 30-days (p = 0.04) than the extracorporeal anastomosis group [16]. Overall operative time in this case was 280 min. Despite a longer operative time reported by many authors, D’Annibale et al. demonstrated that these times significantly decrease with increased surgeon and operative team experience, therefore we support the use of robotic assistance in right colectomy with intracorporeal anastomosis [17]. We performed a Pfannenstiel incision, as usual, for specimen extraction, under vision control to avoid injuries to the VPS catheter, and it was not necessary to manipulate it. In case of no manipulation of the catheter, it is important to confirm fluid flow from the catheter as to monitoring brain pressure and it has been reported that routine anesthesia monitoring alone may be adequate, even though transcranial Doppler was also used for this scope [18,19]. We didn’t use transcranial Doppler, indeed, but only routine monitoring. Some authors argued that subcutaneous seeding or retrograde metastasis due to pneumoperitoneum spread of cancer cells may occur, but it has been widely demonstrated that actual incidence of port-side metastases is not significantly different from wound recurrence after open surgery [20].

Right colectomy for cancer is associated with a lower risk of anastomotic leakage or intraabdominal abscess compared with left colectomy [21]. Patient was discharged on POD 5 and postoperative stay was uneventful, supporting this results in term of short length of stay and lower complication rate after a robotic right colectomy and the safety to perform an intracorporeal anastomosis.

This is the first reported case of a robotic right colectomy with an intracorporeal anastomosis in a patients with a VPS and without manipulation of the shunt catheter. In conclusion, we can consider robotic right colectomy safe also in patients with VPS, with short hospital stay and fast postoperative recovery.

## Conflicts of interest

All the authors declare to have no conflicts of interests.

## Funding

No funds for this manuscript have been received.

## Ethical approval

The current study has been exempt from ethical approval by my Institution.

## Consent

All authors declare to have obtained a written informed consent by the patient.

## Author contribution

I.M., M.L., F.P. have equally contributed to the analysis, interpretation and writing of the paper.

## Registration of research studies

Not available.

## Guarantor

Igor Monsellato, MD, PhD.

## Disclosure statement

No potential conflict of interest was reported by the authors.

## Provenance and peer review

Not commissioned, externally peer-reviewed.

## References

[1] Kanev PM, Park TS. The treatment of hydrocephalus. Neurosurg Clin N Am. 1993;4:611-19. 8241784

[2] Baskin JJ, Vishteh AG, Wesche DE et al. Ventriculoperitoneal shunt failure as a complication of laparoscopic surgery. JSLS. 1998;2:177-80. PMC3015282 9876734

[3] Schwed DA, Edoga JK, McDonnell TE. Ventilatory impairment during laparoscopic cholecystectomy in a patient with a ventriculoperitoneal shunt, J Laparoendosc Surg. 1992;2:57-9. 10.1089/lps.1992.2.57 1576369

[4] Torigoe T, Koui S, Uehara T, Arase K, Nakayama Y, Yamaguchi K. Laparoscopic cecal cancer resection in a patient with a ventriculoperitoneal shunt: A case report. Int J Surg Case Rep. 2013;4(3):330-3. 10.1016/j.ijscr.2013.01.005 PMC3604664 23416501

[5] Ishikawa T, Nishikawa M, Nakamoto H, Yokoyama R, Taketomi A. Laparoscopic anterior resection for rectal cancer in a patient with a ventriculoperitoneal shunt. Asian J Endosc Surg. 2018 Aug;11(3):259-261. 10.1111/ases.12444 29265592

[6] Agha RA, Borrelli MR, Farwana R, Koshy K, Fowler A, Orgill DP, For the SCARE Group. The SCARE 2018 Statement: Updating Consensus Surgical CAse REport (SCARE) Guidelines, International Journal of Surgery 2018;60:132-136. 10.1016/j.ijsu.2018.10.028 30342279

[7] Uzzo RG, Bilsky M, Mininberg DT, et al. Laparoscopic surgery in children with ventriculoperitoneal shunts: Effect pf pneumoperitoneum on intracranial pressure-preliminary experience. Urology. 1993;49:753-57. 10.1016/S0090-4295(97)00233-1 9145983

[8] Schwed DA, Edoga JK, McDonnell TE. Ventilatory impairment during laparoscopic cholecystectomy in a patient with a ventriculoperitoneal shunt. J Laparoendosc Surg 1992;2: 57–59. 10.1089/lps.1992.2.57 1576369

[9] Baskin JJ, Vishteh AG, Wesche DE et al. Ventriculoperitoneal shunt failure as a complication of laparoscopic surgery. JSLS. 1998;2: 177–180. PMC3015282 9876734

[10] Yoshihara T, Tomimaru Y, Noguchi K, et al. Feasibility of laparoscopic cholecystectomy in patients with cerebrospinal fluid shunt. Asian J Endosc Surg. 2017;10:394-98. 10.1111/ases.12380 28387055

[11] Matsumoto T, Endo Y, Uchida H, et al. An examination of safety on laparoscopic surgery in Patients with ventriculoperitoneal shunt by a CO2 reflux experiment. J Laparoendosc Adv Surg Tech. 2010;20(3):231-34. 10.1089/lap.2010.0038 20374011

[12] Wadhwa S, Hanna GK, Barina AR, Audisio RA, Virgo KS, Johnson FE. Gastrointestinal cancer surgery in patients with a prior ventriculoperitoneal shunt: the department of veterans affairs experience. Gastrointestinal Cancer Research. 2012;5:125–9. PMC3433261 23077686

[13] Imagami T, Takayama S, Maeda Y, et al. Laparoscopic Resection of Advanced Colorectal Cancer in a Patient with Lumboperitoneal Shunt. Case Rep Surg. 2018 Nov 14;2018:6826079. 10.1155/2018/6826079 PMC6261403 30538882

[14] Park JS, Kang H, Park SY, et al. Long-term oncologic after robotic versus laparoscopic right colectomy: a prospective randomized study. Surg Endosc. 2018 Nov 19. 10.1007/s00464-018-6563-8 30456502

[15] Ma S, Chen Y, Chen Y, et al. Short-term outcomes of robotic-assisted right colectomy compared with laparoscopic surgery: A systematic review and meta-analysis. Asian J Surg. 2018 Nov 29. 10.1016/j.asjsur.2018.11.002 30503268

[16] Cleary RK, Kassir A, Johnson CS, et al. Intracorporeal versus extracorporeal anastomosis for minimally invasive right colectomy: A multi-center propensity score-matched comparison of outcomes. PLoS One. 2018 Oct 24;13(10):e0206277. 10.1371/journal.pone.0206277 PMC6200279 30356298

[17] D'Annibale A, Pernazza G, Morpurgo E, Monsellato I, et al. Robotic right colon resection: evaluation of first 50 consecutive cases for malignant disease. Ann Surg Oncol. 2010 Nov;17(11):2856-62. 10.1245/s10434-010-1175-0 20567918

[18] Jackman SV, Weingart JD, Kinsman SL, Docimo SG. Laparoscopic surgery in patients with ventriculoperitoneal shunts: safety and monitoring. J Urol. 2000 Oct;164(4):1352-4. 10992414

[19] Ravaoherisoa J, Meyer P, Afriat R et al. Laparoscopic surgery in a patient with ventriculoperitoneal shunt: monitoring of shunt function with transcranial Doppler. British Journal of Anaesthesia. 2004;92(3);434–437. 10.1093/bja/aeh067 14742339

[20] Emoto S, Ishigami H, Yamaguchi H, et al. Port-site metastasis after laparoscopic surgery for gastrointestinal cancer. Surgery Today. 2017;47(3);280–283. 10.1007/s00595-016-1346-0 27226019

[21] Veyrie N, Ata T, Muscari F, Couchard AC, Msika S, Hay JM, et al. Anastomotic leakage after elective right versus left colectomy for cancer: prevalence and independent risk factors. Journal of the American College of Surgeons. 2007;205:785–93. 10.1016/j.jamcollsurg.2007.06.284 18035262

